# In Memoriam

**Published:** 1876-05

**Authors:** 


### IN MEMORIAM.

In the April number of the JOURNAL we promised in this number a more extended notice of the death of the lamented Nottingham. Since then we have received from Drs. Burgess and Green the following biographical sketches. We with pleasure insert both, as we feel that too much cannot be said of one so noble and so good:

DR. C. B. NOTTINGHAM.—Dr. Curtis Bell Nottingham was born in North Hampton county, on the eastern shore of Virginia, May 21st, 1818, and died in Macon, Georgia, 14th of March, 1876, in the 58th year of his age.

His academic education was received at home, under the guardianship of his eldest brother, Mr. Leonard B. Nottingham, and completed in Carlyle College, Pennsylvania. Having determined to make the science of medicine his profession, he worked faithfully to that end, and graduated at Jefferson Medical College, Philadelphia, in 1840.

His professional labors were begun and carried out with marked success for nine years, in Perry, the county site of Houston county, Georgia. Gifted by inheritance with all the attributes of a perfect gentleman, Dr. Nottingham failed not in winning the confidence, admiration, and love of all with whom he was brought in contact. Thus endowed, it is easy to imagine how readily he won the confidence and liberal patronage of the people who delighted to honor him in his new home. For nine years his professional labors grew more extensive and arduous, when he determined to change his field to Macon, in Bibb county.

In this new location was afforded a wider range for the development of a superior order of talent, that was destined to shine with ever-increasing brilliancy to the close of his life. Here he met peers before whom the doubting might feel inclined to shrink. Not so with Dr. Nottingham; with full confidence in his ample qualifications, and an ever-guarded determination to enter upon no other than the legitimate domain of his chosen profession, nothing was more in harmony with his aspirations than to measure strength with an honorable competitor.

In Macon his practice was attended with like successful results as had so signally marked his labors in Houston county. His patronage grew steadily and rapidly, until he stood in the front ranks with the leading men of the profession of Georgia. Naturally of a delicate constitution, the severe tax levied upon him was making perceptible inroads, when he concluded to change his pursuits to the field of agriculture, and to this end moved to Louisiana, in January, 1860. This new enterprise, with the advantages of science to guide, must have yielded abundant fruit, but he was not suffered to remain long in the quiet and uniform repose of the husbandman. The tocsin of war had sounded. It would have been unlike himself had he not at once responded to the calls of duty and his country. With the appointment of surgeon, he was assigned to the supervision of an important military post in the State of his adoption, where he served with characteristic fidelity to the end of the war.

The disastrous result of hostilities, left him, like most of the Southern people whose fortunes consisted in land and negroes, in the midst of poverty. His agricultural prospects thus rudely terminated, he resolved to return to Macon, where he resumed practice on January 1st, 1866, among friends who warmly welcomed him to his former home.

For two or three years after his return his health was quite feeble, but with the exception of a few short intervals when acute disease forced him to his couch, his indomitable energy, and a will that bowed to nothing but the absolute decrees of Deity, bore him triumphantly through a laborious work until the rose-tint of health had been restored to the blanched cheeks. His progress was onward and upward, until he was justly entitled to the first place on the medical staff of the city of Macon.

Some years before his death, his able mind led him to enter successfully the higher domains of gynæcological surgery in successive operations for ovarian tumors. It was the writer's privilege to assist in several of these operations, and with pleasure he bears testimony to the skilful and fearless method which accurate knowledge of his work enabled him to exhibit. The brightness of the dawn in this new field gave abundant evidence of the noon-day splendor just ahead had his life been spared.

Perhaps the most cherished theme of his whole professional life was the inauguration of a State law of registration; and although his immediate effort failed to meet the approbation of a majority of our legislators, whose proceedings, I regret to say, were frequently savored with a disposition to ignore all great measures of public weal, this ideal was at a later day secured in the establishment of a Board of Health for the State, in which was incorporated the registration feature. His Excellency Governor Smith gave to Dr. Nottingham a place on the Board, which was duly appreciated and highly honored by the appointment. His appreciation and full realization of the responsible position he had accepted was evinced by the ceaseless labors bestowed on the work. He was in correspondence with every portion of the continent, and not a leaf was left unturned that could throw light on the subject.

In the writer's opinion, this new work, coupled with his ordinary laborious duties, had much to do with shortening his valuable life. The tax upon mind and body of a constitution naturally frail was too great, and all that was mortal of this truly great and good man perished. Death had no terror to him. Calmly his soul passed into the blessed realms of peace.

*Macon, Ga.* WILLIAM R. BURGESS, M.D.

---

IN MEMORIAM.—Dr. C. B. Nottingham was born on the eastern shore of Virginia May 18th, 1818; moved to Houston county, Georgia, in 1840, and began the practice of medicine. In 1849 he moved to Macon, Georgia, and rapidly built up a large and lucrative practice. In February, 1860, he moved to Northwestern Louisiana, to engage in planting, but his heart yearned for the associations and friendships of his young manhood; and in 1866 we find him again in Macon, where he was welcomed by his old patrons with outstretched arms and open hearts, and soon was engaged in a laborious practice, continuing to the very day he succumbed and took his bed; when, after a lingering and painful illness of eight weeks, on the 15th day of March, 1876, he yielded his noble spirit to the God who gave it, aged fifty-seven years, nine months and twenty-seven days.

This solemn theme is the subject of serious thought to all who were fortunate enough to know this learned, laborious and eminent physician, whose death has so recently cast a gloom over the city and State where he lived and worked. But the sadness of the reflection is mellowed by the fact that the influence of human actions extends beyond the present life of the individual; for the example of the good "lives after them," and blesses the world for generations. Thus it was with our friend, whose name is and will ever continue to be held in the most honorable and affectionate remembrance by all whose privilege it was to know and love him.

The care of those committed to his professional charge and the success of his labors were truly remarkable. At home, in the family circle, he was a most affectionate husband, and indulgent and devoted father; in the community, a good citizen and public benefactor; among his professional brethren, universally loved and esteemed, both for his unifom courtesy and professional learning.

He was the originator of the State Board of Health, and to him is due the credit of its existence. Long and patiently did he work for its creation, and he lived to see it throughly organized; and almost the last act of his life was to write the most learned and interesting paper that has been contributed by any of its members.

His recent paper on Blood Letting, read before the last meeting of the State Medical Association, at Savannah, is a monument of the greatness of his perception and the accuracy of his reasoning, and will be remembered and treasured by the profession for many decades to come.

As a writer, he had the approbation of his greatest contemporaries, who best knew him, and were under no temptation to be partial in his favor. As Dr. Barrow said of the Rev. Richard Baxter, "His practical writings were never mended, and his controversial ones seldom confuted." He cultivated every subject he handled; his expressions were clear and powerful, and convincing to the understanding. In the performance of duty he feared no man's displeasure, nor hoped for any man's preferment. He did not look with contempt on those who stood below him, nor to those who were above him with an envious eye. In his hands every character was safe. He neither flattered the highest nor degraded the lowest, but everyone received that commendation due to his rank and station. By painful study he wasted his valuable strength, searching for knowledge to benefit his fellow-man; and he possessed that which was most desirable in the acquisition of true knowledge, the practical use to which he applied it. He seemed to know and feel that general knowledge, unconnected with practical uses, is good for nothing.

But alas! truly may it be said of my friend, "He that increaseth knowledge, increaseth sorrow." While this is true in this world of sin, not so in the world where he has gone; for there an increase of knowledge will be accompanied with an increase of joy and holy rapture unknown to mortal man. Our friend is no more here on earth; let us who survive him imitate his example and emulate his many virtues. Free from sorrow and death, he mingles with the celestial throng around the throne of the Eternal; and while the pure light of heaven pours upon his immortal intellect, he studies the sublime mysteries of Providence and of grace, and kindles with holy rapture as he contemplates the unfolding perfections of Him who is "all in all."

> "There shall he muse amid the starry glow,
> Or hear the fiery streams of glory flow,
> Or, on the living cars of lightning driven,
> Triumphant, wheel around the plains of heaven."

W. A. Green, M.D.

*Macon, Ga., April 6th,* 1876.

